# Deciphering the Role of Trehalose in Tripartite Symbiosis Among Rhizobia, Arbuscular Mycorrhizal Fungi, and Legumes for Enhancing Abiotic Stress Tolerance in Crop Plants

**DOI:** 10.3389/fmicb.2020.509919

**Published:** 2020-09-17

**Authors:** Mahaveer P. Sharma, Minakshi Grover, Dipanti Chourasiya, Abhishek Bharti, Richa Agnihotri, Hemant S. Maheshwari, Ashwani Pareek, Jeffrey S. Buyer, Sushil K. Sharma, Lukas Schütz, Natarajan Mathimaran, Sneh L. Singla-Pareek, Julie M. Grossman, Davis J. Bagyaraj

**Affiliations:** ^1^Microbiology Section, ICAR-Indian Institute of Soybean Research, Indore, India; ^2^Division of Microbiology, ICAR-Indian Agricultural Research Institute, New Delhi, India; ^3^Stress Physiology and Molecular Biology Laboratory, School of Life Sciences, Jawaharlal Nehru University, New Delhi, India; ^4^Sustainable Agricultural Systems Laboratory, United States Department of Agriculture, Agricultural Research Service, Beltsville Agricultural Research Center, Beltsville, MD, United States; ^5^ICAR-National Institute of Biotic Stress Management, Raipur, India; ^6^Department of Environmental Sciences-Botany, University of Basel, Basel, Switzerland; ^7^M S Swaminathan Research Foundation, Chennai, India; ^8^Plant Stress Biology, International Centre for Genetic Engineering and Biotechnology, New Delhi, India; ^9^Department of Horticultural Science, College of Food, Agricultural and Natural Resource Sciences, University of Minnesota, St. Paul, MN, United States; ^10^Center for Natural Biological Resources and Community Development, Bengaluru, India

**Keywords:** trehalose, arbuscular mycorrhizal fungi, rhizobia, legumes, drought stress

## Abstract

Drought is a critical factor limiting the productivity of legumes worldwide. Legumes can enter into a unique tripartite symbiotic relationship with root-nodulating bacteria of genera *Rhizobium*, *Bradyrhizobium*, or *Sinorhizobium* and colonization by arbuscular mycorrhizal fungi (AMF). Rhizobial symbiosis provides nitrogen necessary for growth. AMF symbiosis enhances uptake of diffusion-limited nutrients such as P, Zn, Cu, etc., and also water from the soil *via* plant-associated fungal hyphae. Rhizobial and AMF symbioses can act synergistically in promoting plant growth and fitness, resulting in overall yield benefits under drought stress. One of the approaches that rhizobia use to survive under stress is the accumulation of compatible solutes, or osmolytes, such as trehalose. Trehalose is a non-reducing disaccharide and an osmolyte reported to accumulate in a range of organisms. High accumulation of trehalose in bacteroids during nodulation protects cells and proteins from osmotic shock, desiccation, and heat under drought stress. Manipulation of trehalose cell concentrations has been directly correlated with stress response in plants and other organisms, including AMF. However, the role of this compound in the tripartite symbiotic relationship is not fully explored. This review describes the biological importance and the role of trehalose in the tripartite symbiosis between plants, rhizobia, and AMF. In particular, we review the physiological functions and the molecular investigations of trehalose carried out using omics-based approaches. This review will pave the way for future studies investigating possible metabolic engineering of this biomolecule for enhancing abiotic stress tolerance in plants.

## Introduction

Global climate change is projected to increase average temperatures, change rainfall patterns, and increase water scarcity (Karl and Trenberth, 2003). These effects will be felt especially in the semi-arid tropics, where evaporation and temperature are already high (Vadez et al., 2012). Among all abiotic stresses, drought (water deficit) has been identified as a critical factor limiting crop productivity, with roughly 64% of global land area affected (Meena et al., 2017). Abiotic stresses cause changes in the soil–plant–atmosphere continuum, resulting in crop yield reductions. Prolonged exposure to drought leads to altered metabolism and damage to biomolecules (Bhagat et al., 2014). Legumes deliver several vital environmental, economic, and social services and are a major source of food, nutrition, and feed worldwide, particularly in marginal regions of the global south. They are an essential component of the N cycle, with most species forming symbiotic relationships with diazotrophic bacteria (i.e., rhizobia). Legumes enrich agricultural systems with biologically fixed atmospheric N through the process of biological N fixation (BNF), reducing dependence on chemically produced nitrogenous fertilizers. BNF contributes 40–80% of N worldwide under different agronomic practices (Herridge et al., 2008), where 110–220 kg N/ha/year is fixed by perennial legumes, and 50% of this range is fixed by annual legumes (Havlin et al., 2005). Symbiotic BNF in legumes is highly sensitive to abiotic stresses such as drought and salinity. The protective mechanisms evolved by plants to adapt to stress include the upregulation of compatible solutes, e.g., osmoprotectants, osmolytes, and the activation of both enzymatic and non-enzymatic defense sites.

The rhizosphere is the zone of enhanced microbial activity because of the supply of nutrients *via* host plant root exudates. Plant-growth promoting rhizobacteria, especially rhizobia, colonize plant cells within root nodules, whereas arbuscular mycorrhizal fungi (AMF) form highly branched structures called arbuscules inside the root cortex, which acts as nutrient exchange site between the plant and AMF. The interaction of these two symbiotic entities with the plant helps the host plant with nutrition and protection against soil-borne pathogens (Vessey, 2003; Venant et al., 2013). The tripartite symbioses among rhizobia, AMF, and plants demonstrate the complexity of microbial interactions, resulting in enhanced resistance of plants to environmental stresses (Antunes and Goss, 2005; Antunes et al., 2006). AMF colonize the roots of 80% of terrestrial plant species and positively influence plant growth by augmenting soil nutrient transport, particularly P, N, Zn, and Cu (Govindarajulu et al., 2005). N-fixing rhizobia nodulate most legumes and enhance plant growth by fixing atmospheric N (Baral et al., 2016).

The symbiotic association of leguminous plants with trehalose-producing rhizobia has been reported to impart abiotic stress tolerance to the plants (Suárez et al., 2008; Sugawara et al., 2010; Garg and Pandey, 2016). Species of rhizobia and host genotype play a key role in the determination of the extent of trehalose accumulation by their symbiotic partner and can result in enhanced BNF. Inoculation of *Rhizobium etli* strains, characterized by the overexpression of trehalose-6-phosphate synthase into *Phaseolus vulgaris*, has been shown to enhance both the number of root nodules and N fixation parameters (Suárez et al., 2008).

Trehalose acts as a chemical chaperone as well as a metabolite for preventing the protein from acetylation and glycation under desiccation stress. High glucose in bacterial cells leads to acetylated aggregates, vitrification, and advanced glycation end products for cross-link formation of the proteins (Laskowska and Kuczyñska-Wiśnik, 2019). Trehalose accumulation in *Rhizobium leguminosarum* bv. *trifoli* strain NZP561 occurs by both otsA and TreYZ pathways during the stationary phase. It has been deemed essential for desiccation tolerance and overcoming stress related to nodule occupancy (McIntyre et al., 2007). Similarly, trehalose biosynthesis in *Bradyrhizobium japonicum* occurs by *OtsA*, *TreS*, and *TreY* genes playing a role in symbiotic N-fixation and survivability under salinity (Sugawara et al., 2010).

Mycorrhizal interaction can also impart benefits to trehalose production. Inoculation of the legume *Cajanus cajan* with the AMF (*Rhizophagus irregularis*), in combination with rhizobia, improved nodulation, N and phosphorus (P) uptake, and accumulation of higher trehalose in plants under salinity stress. The higher trehalose content was attributed to the increased activity of terpene synthase enzyme and decreased level of trehalase (Garg and Pandey, 2016). Furthermore, combined AMF with silicon inoculation in *C. cajan* genotypes has been shown to help plants to survive under cadmium and zinc heavy metal stress by improving rhizobial symbiosis competency and regenerating nodules. This was attributed to trehalose synthesis, phytochelatin synthesis, and reactive oxygen species (ROS) scavenging mechanisms (Garg and Singh, 2018). Similarly, the inoculation of AMF and polyamines along with *Sinorhizobium fredii* strain AR-4 was found to counteract nickel toxicity by increasing nodule functioning and modulating trehalose and ureide metabolism (Garg and Saroy, 2020).

Bacteria accumulate osmoprotective solutes, such as trehalose, in response to osmotic or desiccation stress (Vriezen et al., 2007). The survival of trehalose-loaded cells was found to be better than that of non-loaded cells when soybean (*Glycine max*) seeds were coated with cells and subjected to desiccation (Streeter, 2003). Trehalose accumulation in cultured cells and bacteroids by *B. japonicum* (Streeter, 1985) and subsequent increase due to desiccation stress have been reported (Cytryn et al., 2007), suggesting trehalose’s role as an osmoprotectant. The enhanced survival of *B. japonicum* in response to desiccation and salinity stress after exogenous addition of trehalose has been reported (Streeter, 2003). Ocón et al. (2007) investigated the role of trehalose in AMF-mediated plants in which they correlated gene expression and the enzymes involved in trehalose metabolism with AMF hyphal biomass. AMF-mediated plants with lower doses of external trehalose application have been shown to induce both biotic and abiotic stress-related genes, mainly promoting downregulated abiotic stress-associated genes (Schluepmann et al., 2004). Additionally, the roles of various bacterial-mediated trehalose biosynthetic pathways, their relationship with physiological responses, and their expression of stress tolerance genes have been studied (Sugawara et al., 2010). However, no systematic studies on trehalose accumulation and mobilization by the tripartite association of plants with rhizobia and AMF have been carried out. In this review, we discuss the significance of tripartite symbiosis of plants, rhizobia, and AMF in trehalose accumulation, metabolism, genomics, and their importance for mitigating abiotic stresses.

## Trehalose in Plants for Abiotic Stress Tolerance

Trehalose is a non-reducing disaccharide (α-D-glucopyranosyl-1 and 1-α-D-glucopyranoside, comprising two α-glucose molecules) found in plants, insects, invertebrates, and microorganisms. The two α-glucose molecules joined by a glycosidic bond store a low level of energy, which imparts it a more stable configuration than sucrose (Iturriaga et al., 2009). Trehalose is a “minor” sugar produced in plants in response to biotic and abiotic stresses, as part of the mitigation mechanisms. Trehalose is considered a double-faced molecule, which, on the one hand, is required for the infectivity of many pathogens but, at the same time, also elicits plant defense against stress (Fernandez et al., 2010).

Trehalose was reported for the first time in soybean (*G. max*) root nodules during the early 1980s and was found to play a protective role especially during abiotic stress; however, the exact role is still unclear (Goddjin and van Dun, 1999; Fernandez-Aunián et al., 2010). In plants, the possibility exists of developing genotypes with stress-protective capacity by the enhanced expression of trehalose synthase genes or by using genetically engineered bacteria or mutants with improved capacity for accumulating higher trehalose as bio-inoculants. One example is rice cultivar R-64 which, through genetic modification and overexpression of trehalose-synthesizing trehalose-6-phosphate synthase (TPS) and trehalose-6-phosphate phosphatase (TPP) genes and fusion constructs trehalose-6-phosphate synthase/phosphatase (TPSP) from *Escherichia coli*, could tolerate multiple stresses like drought and salinity. The transgenic rice plants showed higher chlorophyll content, low sodium accumulation, high grain yield, and high trehalose accumulation (Joshi et al., 2020). Trehalose is a non-toxic carbon molecule characterized by its ability to accumulate at high concentrations in the cytoplasm and maintain turgidity besides its ability to evoke plant defense mechanisms (Schluepmann et al., 2004). During dehydration, the enhanced trehalose concentration acts as a protector for both proteins and membranes and also stabilizes metabolism in plants (Goddjin and van Dun, 1999; Liu et al., 2008). Moreover, trehalose-accumulating plants trigger ROS and guard macromolecular structures from the destabilizing effect of dehydration occurring during anhydrobiotic situations (Mittler et al., 2004). Bharti et al. (2016) identified soybean genotypes accumulating trehalose in root nodules formed by native rhizobia, which were recovered and characterized. However, improved mechanistic explanations for the correlation between trehalose concentrations and the physiological traits involved in abiotic stress tolerance are needed. Highly trehalose-mediated plants (either mutant or genetically modified) show membrane stability, regulate osmolytes, and have better tolerance to stresses when exposed to stress such as drought or salt (Romero et al., 1997). Exogenous trehalose application in tomato seedling stressed with salinity lowers starch content and increases soluble sugar content and distribution of sugars by modulating sugar-metabolism-related genes and sugar-transport-related genes, respectively. Trehalose application modulates the expression of abscisic acid (ABA)-metabolism-related genes like NCED1, NCED2, CYP707A1, and CYP707A2 for increasing ABA content to alleviate salt tolerance (Feng et al., 2019). Nevertheless, besides using trehalose as stress-protecting carbohydrates in microbial formulations, the exogenous uses (forms, mode, and doses) for application in legume plants need thorough investigation.

## Trehalose in Microbial Abiotic Stress Tolerance

Several researchers have reported improvement in the survival of bacterial cells due to trehalose accumulation. Streeter (1985) observed trehalose accumulation in cultured bacteria of *B. japonicum* under low O_2_ conditions, indicating that trehalose formation in nodules may be induced by a microaerophilic environment. Interestingly, these bacteria cannot grow using trehalose as the exclusive carbon (C) source, suggesting a function other than an energy source. The exogenous application of trehalose to bacterial cultures increased the accumulation of trehalose and improved their survival during the subsequent drying period (Leslie et al., 1995; Streeter, 2003). In soybean seeds, to which trehalose was co-inoculated with *B. japonicum*, this resulted in a threefold increase in trehalose concentration in *B. japonicum* cells and the increased survival of cells on seed surfaces. After 24 h of desiccation, a 294% increase in cell survival was reported when bacteria were grown in medium supplemented with trehalose, indicating a correlation between trehalose concentration and bacterial survival. Furthermore, for the survival of *B. japonicum* during desiccation, the accumulation of trehalose was found to be more critical in the cytoplasm than in the periplasmic space. Similarly, in free-living *R. etli*, overexpression of the OtsA gene encoding TPS led to an increased tolerance of osmotic stress (Suárez et al., 2008), whereas its mutation otherwise impaired stress tolerance. A macroarray analysis of 7,200 expressed sequence tags from legume nodules inoculated with a rhizobia strain overexpressing trehalose-6-phosphate synthase gene revealed the upregulation of genes involved in stress tolerance and C and N metabolism, suggesting a signaling mechanism for trehalose. In *B. japonicum*, the accumulation of trehalose also occurs as a consequence of salt-induced osmotic stress. The mutant strains without trehalose biosynthetic genes failed to grow on medium supplemented with salt (Domínguez-Ferreras et al., 2009; Sugawara et al., 2010). In another study, four trehalose-accumulating rhizobial strains isolated from the root nodules of *P. vulgaris* were shown to have improved recovery from salt conditions (Fernandez-Aunián et al., 2010). These studies indicate rhizobial trehalose metabolism to play a key role in signaling plant growth, yield, and adaptation to abiotic stress, and its manipulation implies having a tremendous agronomic impact on leguminous plants (Suárez et al., 2008).

## Rhizobia-Mediated Trehalose Metabolism and Its Role in Abiotic Stress Tolerance

Agricultural productivity benefits from the rhizobia–legume symbiotic association. It is well understood that trehalose sugar acts as a strategy to combat abiotic stress, where dehydrated enzymes and membranes are stabilized by trehalose and protect against desiccation of structures. Healthy plants generally do not produce adequate trehalose concentrations to induce osmoprotectant benefits. This does not impede trehalose gene expression and biosynthesis, and a very low concentration of trehalose has been reported in some plants (Leyman et al., 2001). Some plants have enhanced concentrations as a result of colonization by AMF (Schubert et al., 1992) and N-fixing rhizobia (Streeter, 1985). By recording the ^1^HNMR spectra of extracellular cultures, Montes-Grajales et al. (2019) identified trehalose in a culture supernatant of N-fixing rhizobial strains *R. etli* CFN42 and *Rhizobium leguminosarum phaseoli* Ch24-10. Under symbiotic conditions, trehalose is synthesized by the bacteroid in the nodule; however, the majority of trehalose is localized in the cytoplasm of host plant cells (Farias-Rodriguez et al., 1998). Low concentrations of trehalose have also been detected in aboveground legume biomass, indicating translocation (Streeter, 1980). Trehalose concentrations are variable in bacteroids and nodules of numerous leguminous plant species, with substantial quantities reported in soybean nodules when the nodules are in terminal senescence (Müller et al., 2001). Rhizobial strain genetics and plant age have been shown to impact the degree to which trehalose is accumulated in bacteroids (Streeter, 1985).

Trehalose synthesis in most rhizobial species takes place both in free-living and symbiotic phases and has the functional significance of disaccharide production (Jensen et al., 2005; Streeter and Gomez, 2006). Trehalose biosynthesis in symbiotic N-fixing bacteria occurs as a result of three distinct pathways, with *B. japonicum* and *Bradyrhizobium elkanii*, each having the three completely independent mechanisms for synthesis (Streeter and Gomez, 2006). The work of Streeter and Gomez reports the role of trehalose in the protection against desiccation, identifying three enzymatic drivers including trehalose synthase (TS-*TreS*), maltooligosyl trehalose synthase (MOTS-*TreY*), and trehalose-6-phosphate synthetase (TPS-*OtsA*). It was found that the bacteroids of nodules contained a higher concentration of TS nodule enzyme in comparison to the substrate for TS, maltose. This could be due to the high trehalose activity in bacteroids, inhibiting TS, and MOTS in bacteroids. However, in the family *Rhizobiaceae*, most *Rhizobium* species synthesize trehalose, except in the species *Rhizobium tropici* (Streeter and Gomez, 2006). Nevertheless, Domínguez-Ferreras et al. (2009) concluded that, for competitive and effective nodulation of alfalfa by *Sinorhizobium meliloti*, the trehalose biosynthesis pathway is essential. Upon further examination of the presence of genes encoding for the three pathways of trehalose synthesis, i.e., *OtsA*, *TreYZ*, and *TreS* in *S. meliloti* and *R. etli*, *R. etli* was found to have an overexpression of *OtsA* gene in the presence of osmotic stress, which increased its tolerance to osmotic stress and likely provided the inoculant with a higher survival capacity and symbiotic efficiency (Suárez et al., 2008). To date, in *R. etli*, three genes, *TreYZ*, *TreS*, and *OtsAB*, are present and involved in the trehalose biosynthesis pathway and exhibit a transcriptional response toward osmotic stress. *OtsA* was found to have a major role (with higher activity in the cultured cell than the symbiotic bacteroids) in trehalose accumulation and osmoadaptation. This indicates the upregulation of enzymes involved in trehalose synthesis and downregulation of others when a symbiotic relationship with the soybean plant is initiated by *Bradyrhizobium*. Quantitative reverse transcription-polymerase chain reaction (RT-PCR) analysis shows the significant upregulation of the *OtsA* gene along with *nodC*, *P5CR*, and molecular chaperone genes in *Mesorhizobium ciceri* cells incubated under polyethylene glycol (PEG)-induced desiccating stress conditions, indicating the prominent role of trehalose in *M. ciceri* drought stress tolerance (Yadav et al., 2020).

It is now well established that rhizobia are capable of synthesizing trehalose, which accumulates in nodules and bacteroids (Müller et al., 2001). The accumulation of trehalose in root nodules is chiefly determined by three factors, namely, rhizobia strain genotype, legume genotype, and the surrounding environment (Farias-Rodriguez et al., 1998). Trehalose accumulation in bradyrhizobial cells promotes nodulation, with soybean acting as a compatible solute to overcome host-induced osmotic stress that occurs during nodulation (Sugawara et al., 2010). Conversely, the accumulation of trehalose in *R. leguminosarum* and *S. meliloti* did not increase the symbiotic effectiveness on clover and alfalfa genotypes (Ampomah et al., 2008). This suggests that nodule occupancy and colonization ability may vary across alfalfa and clover cultivars, with the bacterial genotypes studied varying with trehalose utilization mutants of *S. meliloti* and *Sinorhizobium medicae* (*thuB*) and their parent strains (Ampomah et al., 2008). It has also been reported that, when applied exogenously to *S. meliloti*, *R*. *leguminosarum* bv. *trifolii*, and *R. leguminosarum* bv. *phaseoli*, trehalose may act as an osmoprotectant; however, observations indicate an indirect contribution of trehalose to cell turgor resulting from a stimulated increase in the osmolyte levels of glutamate and *N*-acetylglutaminyl glutamine amide rather than the increased cell concentrations (Gouffi et al., 1998).

Räsänen et al. (2004) evaluated the role of trehalose in the symbiosis of *Acacia senegal*–*Sinorhizobium* under drought stress and found that trehalose (0.01, 0.05, and 0.09 M) led to osmotic stress protection (9 and 17% PEG). Furthermore, when trehalose (applied exogenously at 0.0003 M in soil) was exposed to severe drought stress and planted with *A. senegal*, the plant was able to maintain even higher numbers of culturable rhizobia than under moderate drought. They concluded that a basal amount of trehalose is likely to be present in the nodules even under unstressed conditions, but under drought conditions the trehalose levels increase significantly (Räsänen et al., 2004). Another study by Song et al. (2017), based on gas chromatography–time-of-flight mass spectrometry analysis, reported over 20-fold increase in trehalose concentration in the roots of *Rhizobium*-treated plants after 6 days of alkali stress, while no such changes were observed in non-inoculated plants. These results indicate that trehalose may be affected by alkali stress in plants with root nodule symbiosis. A metabolomic study was performed to identify the compounds produced in roots and root hairs during the nodulation of soybean by *B. japonicum*. Of the 166 metabolites significantly regulated in response to *B. japonicum* inoculation, trehalose was among the most strongly induced metabolites produced following inoculation. Subsequent metabolomic analyses of root hairs inoculated with a *B. japonicum* mutant defective in trehalose synthase (trehalose 6-phosphate synthase) and maltooligo-syltrehalose synthase genes (*ΔotsA ΔtreS ΔtreY* triple mutant) showed that the trehalose detected in the inoculated root hairs was primarily of bacterial origin. During the infection process, *B. japonicum* may experience osmotic stress either on the root hair surface or within the infection thread, and trehalose might be playing a role in protecting actively nodulating roots against drought and other stress factors (Brechenmacher et al., 2010). The enhanced levels of trehalose in root nodules may also protect bacterial nitrogenase activity under moisture stress conditions (Jiménez-Zacarías et al., 2004). In addition, the increased concentrations of nodule trehalose have been correlated to increased BNF efficiency, which consequently improved the drought and salinity tolerance of the plant (Jiménez-Zacarías et al., 2004; López et al., 2008). In *R. etli*, mannitol is a precursor for trehalose synthesis, but the bacterial strain genome does not encode specific mannitol phosphotransferase as it does not mediate the entry of mannitol into the cell. Hence, it is likely that *smoEFGK* (which encodes a sorbitol/mannitol ABC transporter), *mtlK* (which encodes a mannitol 2-dehydrogenase transporter which transforms mannitol to fructose), and xylA (which encodes a xylose isomerase that transforms fructose to glucose) are involved (Reina-Bueno et al., 2012). This suggests that, during the initial stages of symbiotic interactions, the host drives trehalose production, underscoring its capacity to mitigate plant-induced stress during infection instead of influencing the colonizing ability of roots, which is critical for competitiveness.

## AMF-Mediated Trehalose Metabolism and Dynamics in the Association With Plants

Plant benefits derived from AMF colonization are desirable for sustainable agricultural management strategies with the reduction of synthetic fertilizers (Douds et al., 2010). AMF is dependent on their plant partners for carbohydrates, where both glycogen and trehalose serve as the chief carbohydrate sources (Bécard et al., 1991), stored mainly (up to 95%) as triacylglycerols (TAGs). In AMF, trehalose is stored as C in the extraradical hyphae and spores of AMF (Bécard et al., 1991; Bago et al., 2003). Colonized plants deliver sucrose to the roots and enzymatically hydrolyze sucrose to maintain a pool of hexoses available for transfer to the fungus. In AMF, the alkaline invertase enzyme then converts hexoses to trehaloses (Smith and Read, 2008). These fungi, with the help of intraradical hyphae, draw C in the form of hexoses, which are then rapidly converted to glycogen and trehalose and then to lipids (Shachar-Hill et al., 1995; Pfeffer et al., 1999). To improve the plant’s stress tolerance, trehalose must then be transported back to the plant using specialized transporters in the membrane designed to facilitate hexoses and lipids. This suggests that trehalose accumulation in AMF may play a more significant role in cellular stress protection than previously thought. However, the isolation of neither trehalose synthesis genes nor their encoding enzymes has been reported in AMF. Lipids in the external mycelium of AMF are also converted to hexoses and trehalose (Ocón et al., 2007), with the role of trehalose in AMF implicated as intermediate carbon storage (Bécard et al., 1991; Bago et al., 2003). It has been shown that trehalose levels increase under stress, possibly as an energy source (Delorge et al., 2014). Schubert et al. (1992) showed that the trehalose content in *Glomus mosseae* (currently known as *Funneliformis mosseae*)-inoculated soybean roots increased positively with the extent of fungal colonization and decreased with P fertilization and dark conditions. Trehalose levels have also been shown to increase under heat stress as well as chemical stress imposed through arsenate (Ocón et al., 2007). Information on the extent to which trehalose is induced under stress across AMF genotypes is not available.

## Omics-Based Studies in AMF Concerning Trehalose Metabolism

The first genome sequence of an AMF was published in 2013 for the model strain *R. irregularis* DAOM197198 (Tisserant et al., 2013). To date, complete genome sequences are available for only two AMF from a single genus, *Rhizophagus* (Kobayashi et al., 2018). These studies have been instrumental as they provided the first indication that AM fungi are fatty acid auxotrophs (Wewer et al., 2014). The genome of these two organisms contains ∼579–739 protein-encoding genes, and very interestingly, the loss of several genes that are otherwise present in their close relatives has also been reported (Huerta-Cepas et al., 2015). In the genome of *Rhizophagus clarus*, the absence of cytosolic fatty acid synthase (FAS) was noted, whereas all mitochondrial FAS components were present (Kobayashi et al., 2018). In another study, the transcriptomic analysis of sunflower and AMF revealed that differentially expressed genes (DEGs) were explicitly involved in known mycorrhizal processes, such as membrane transport and cell wall shaping. The most critical DEGs were carefully described to hypothesize their role in AM symbiosis (Vangelisti et al., 2018). Studies on the analyses of gene expression in AM-mediated roots revealed changes both in the plant and fungal transcriptomes, which are linked to mycorrhizal establishment and development (Shu et al., 2016). However, not much work has been carried out on AMF genomics concerning trehalose metabolism.

The alignment of *G. mosseae* and *Glomus intraradices* (currently known as *R. irregularis*) NTH1 with 21 other neutral trehalases showed that the AM fungal proteins are highly conserved in their protein sequence and structures (Ocón et al., 2007). The EF-like Ca^2+^-binding site localized at the N-terminus of both AM fungal proteins has also been identified in all fungal neutral trehalases (Ocón et al., 2007). This motif could contribute to the further regulation of enzyme activity as has been proposed for other fungi (Franco et al., 2003). Studies on the structures *in vivo* and biochemical studies have revealed the significance of the Tps2 N-terminal domain in fungal cellular stress responses and the conformational flexibility of the Tps2 C-terminal domain that imposes exquisite substrate specificity and permits efficient catalysis. These structures pave the way for “rational” inhibitor design against Tps2, facilitating antifungal drug design (Miao et al., 2016).

Assessment of trehalose content along with its transcriptional regulation and enzyme activity (for neutral trehalase and trehalose-6-phosphate phosphatase) was carried out in *G. intraradices* in response to heat shock and chemical or osmotic stress (Ocón et al., 2007). Prolonged or intensive exposure to heat or chemical stress, but not osmotic stress, caused an increase in trehalose in the hyphae of *G. intraradices.* The transient upregulation of the trehalose-6-P phosphatase (GiTPS2) transcript coincided with the rise in enzyme activity (Ocón et al., 2007). However, there were no changes in neutral trehalase (GiNTH1) RNA accumulation in response to the imposed stress (Ocón et al., 2007).

## Tripartite Symbiosis and Abiotic Stress Tolerance

The formation of a tripartite symbiosis requires nodule-forming soil microbes, including but not limited to *Rhizobium*, *Bradyrhizobium*, *Sinorhizobium*, *Mesorhizobium*, and *Azorhizobium* (Hirsch, 1992) and AMF (Koide and Schreiner, 1992). Both rhizobial and fungal symbiotic partners accelerate mineral nutrient acquisition in exchange with carbon from the host. N and P are essential worldwide for plant growth, with poor soils requiring frequent amendments. During the tripartite relationship, rhizobia and AMF appear to act synergistically, with this combined inoculation enhancing plant growth and N content compared to single inoculation with either *Glomus* or *Rhizobium* (Bagyaraj et al., 1979) and leading to improved host colonization by both inoculants (Van Der Heijden et al., 2015). Although some studies on rhizobial–legume symbiosis have shown that, under drought stress, an increase in trehalose content was accompanied by an increase in plant stress tolerance, reports of trehalose accumulation in mycorrhiza are scarce, where, unlike rhizobia, plants do not receive carbohydrate from the microsymbiont. Moreover, the role of trehalose in the tripartite symbiosis of rhizobia–mycorrhiza–legume needs a detailed study. Combined mycorrhizal colonization and rhizobial nodulation have been reported to increase legume tolerance to drought. Ruiz-Lozano et al. (2001) observed that soybean plants inoculated with AMF could cope better with premature nodule senescence induced by drought stress. In faba bean (*Vicia faba*) under drought conditions, inoculation with mycorrhiza was shown to enhance nodulation in comparison to non-mycorrhizal plants (Abdel-Fattah et al., 2002). Under drought stress, inoculation with *G. mosseae* caused an increase in P uptake by maize plants in comparison to an uninoculated plant (Abdelmoneim et al., 2014).

Arbuscular mycorrhizal fungi, in combination with rhizobia, are also considered as optimal for the reclamation of saline soils. Under salt stress, a substantial improvement in plant biomass and N-fixing ability of nodules due to the symbiotic relationship between *C. cajan* and *G. mosseae* has been reported. Although the response was plant genotype dependent, AMF symbiosis could improve the plant’s ability to survive under salt stress by enhancing mineral nutrient acquisition (P, Zn, Fe), water use efficiency, rate of photosynthesis, and accumulation of trehalose (Garg and Chandel, 2011).

Ballesteros-Almanza et al. (2010) explored the role of tripartite symbiosis on trehalose accumulation in the nodules of three wild genotypes of common bean, two commercial genotypes of *P. vulgaris* inoculated with *G. intraradices*, and seven native AMF species planted at different moisture regimes. They found that co-inoculation with both microsymbionts negatively affected trehalose accumulation in the nodules of each genotype as compared to single inoculation either with *Rhizobium* or AMF, both under normal as well as stressed conditions. Overall, AMF colonization maintained a significant positive correlation with trehalose content in the nodules. These reports indicate a definite role of trehalose in BNF in mycorrhizal legume plants under abiotic stress conditions. Garg and Saroy (2020) investigated the role of AMF in improving rhizobial symbiosis and trehalose metabolism under nickel (Ni) stress in *C. cajan*. AMF inoculation was found to enhance nodulation, N-fixing potential, and trehalose synthesis under Ni toxicity. Due to the high activity of the trehalase (TRE) enzyme, a very low level of trehalose was observed in the nodules under control conditions. However, Ni stress reduced the TRE activity and thus increased the trehalose levels to a certain extent. Interestingly, a negative correlation between trehalose and nitrogenase activity indicated that nodules with reduced nitrogenase activity under Ni stress tended to synthesize more trehalose, suggesting that more trehalose is synthesized because of low demand for reduced C in the bacteroids. The antioxidant effect of trehalose might also protect nitrogenase from inactivation. Moreover, a significant decline in TRE activity was recorded with AMF inoculation, which resulted in significantly improved trehalose concentrations in the nodules. This increment in trehalose, accompanied by the increased activity of T6PS and T6PP enzymes, indicated a direct role of AMF in upregulating trehalose biosynthesis in pigeon pea plants to overcome Ni stress.

It has been shown that the AMF community composition not only differs between legume and non-legume plants but also is impacted by crop sequences in rotation. Distinct AMF species and strains have been identified in the root nodules of legumes compared to non-legume roots (Scheublin et al., 2004). It is, therefore, necessary to cultivate AMF and rhizobial strains from the same niche of the rhizosphere of that legume–crop sequence. A list of examples on tripartite symbiosis-mediated abiotic stress tolerance in legume plants is provided in Table 1.

**TABLE 1:** Examples of AM fungi-rhizobia mediated stress tolerance in legume plants*.

Stress	Microorganism	Plant species	Inference	References
Heat shock and osmotic stress	*Glomus intraradices*	AM extraradical hyphae in *in vitro* system	Increase trehalose turnover rate	Ocón et al. (2007)
Drought	*Septoglomus constrictum*, *Glomus* sp., and *Glomus aggregatum*	*Glycine max*	Improved water content and P and N concentrations	Grümberg et al. (2015)
Drought	*Glomus intraradices* and native rhizobial strains	Genotypes of *Phaseolus vulgaris*	Showed positive correlation between mycorrhizal colonization and nodule trehalose content	Ballesteros-Almanza et al. (2010)
Drought	Trehalose	*Acacia senegal*, *Sinorhizobium* symbiosis	Protected cell cultures of the *Sinorhizobium* strains and increased EPS	Räsänen et al. (2004)
Osmotic stress	*Glomus intraradices* BEG 123	*Phaseolus vulgaris*	Enhanced osmotic root hydraulic conductance in roots through active solute transport	Aroca et al. (2007)
Salt	*Glomus etunicatum*	*Glycine max*	Influenced proline concentrations	Sharifi et al. (2007)
Drought	Co-inoculation of *Rhizobium tropici* and *Paenibacillus polymyxa*	*Phaseolus vulgaris*	Upregulation of genes involved in stress tolerance	Figueiredo et al. (2008)
Salt	*Glomus intraradices*	*Glycine max*	Accumulation of carbohydrates	Porcel and Ruiz-Lozano (2004)
Salinity	*Glomus intraradices* BAFC 3108	*Lotus glaber*	Reduced sodium in root and shoots; enhanced root potassium	Sannazzaro et al. (2006)
Salinity	*Glomus clarum*	*Vigna radiata*		Rabie et al. (2005), Daei et al. (2009)
Salinity	Glomus intraradices BEG121	*Lactuca sativa*	Reduced concentration of ABA	Aroca et al. (2008)
Salt	Native rhizobia	*Medicago truncatula* and *Phaseolus vulgaris*	Enhanced trehalose	López et al. (2008)
Salt	*Glomus mosseae*	*Cajanus cajan*	Enhanced trehalose in nodules	Garg and Chandel (2011)
Salt	*Funneliformis mosseae Rhizophagus irregulariae*		Enhanced trehalose turnover in AM plants, nodulation and N-fixation	Garg and Pandey (2016)

*Modified from Meena et al. (2017).

## Raising High-Trehalose-Accumulation Plants

**FIGURE 1:**
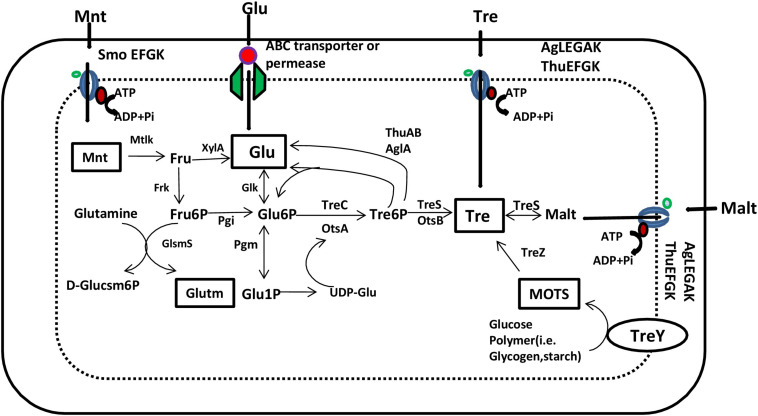
Scheme of trehalose metabolism in *Rhizobium etli* based on the annotated genome. Glu, D-glucose; Glu6P, D-glucose-6-phosphate; Glu1P, D-glucose-1-phosphate; Glutm, D-glutamate; D-Glucsm6P, D-glucosamine-6-phosphate; Fru, D-fructose; Fru6P, D-fructose-6-phosphate; Malt, maltose; Mnt, mannitol; MOTS, maltoolygosyltrehalose; Tre, trehalose; TreP, trehalose-6-phosphate; AlgEFGAK and ThuEFGK, putative trehalose/maltose/sucrose ABC transporters; GlmS, glucosamine-6-phosphate synthase; Mtlk, mannitol 2-dehydrogenase; Frk, fructokinase; OtsA, trehalose-6-phosphate synthase; OtsB, trehalose-6-phosphate phosphatase; Pgi, phosphoglucose isomerase; XylA, xylose isomerase; TreC, trehalose-6-phosphate hydrolase; TreS, trehalose synthase; TreY, maltooligosyl trehalose synthase; TreZ, maltooligosyl trehalose trehalohydrolase; SmoEFGK, sorbitol/mannitol ABC transporter (source: Reina-Bueno et al., 2012).

Approaches such as breeding for trehalose-over-producing genotypes and genetically engineering the plants for trehalose over-production are strategies for improving trehalose accumulation for abiotic stress tolerance. Transgenic varieties of tobacco, *Arabidopsis*, potato, alfalfa, rice, and tomato plants tolerant to diverse abiotic stresses have been developed by transformation with yeast/bacterial *TPS/OtsA* genes (Iturriaga et al., 2009). Initially, plasmid p42e (*treY*) and the chromosome and plasmid p42f (two copies of *treZ*) were reported to contain the genes responsible for trehalose synthesis. Reina-Bueno et al. (2012) provided a genomic analysis of the trehalose metabolism pathway in *R. etli*, illustrating the genes involved in trehalose synthesis, transport, and degradation (Figure 1).

Tolerance to abiotic stress, particularly drought, was observed even when trehalose accumulated at lower concentrations in transgenic plants designed to overexpress microbial trehalose biosynthetic genes. Drawbacks of such studies include possible growth aberrations of the transgenic plants due to the accumulation of trehalose-6-phosphate, which plays a critical role in gene regulation during the seedling stage of development (Chary et al., 2008). Another approach includes the use of trehalose-over-producing mutant microorganisms as bio-inoculants to ease abiotic stresses in plants. Suárez et al. (2008) reported a significant increase in nodules inoculated with a trehalose-overproducing strain of *R. etli*, which resulted in a substantial increase in the expression of leghemoglobin-2 gene. This suggests the possibility of increased nitrogenase activity, N fixation, and bacteroid respiration. These observations indicated that the trehalose over-expressing strain of *R. etli* associated with common bean plants significantly fixed N and increased the nodule number, biomass, and grain yield. This hypothesis is further supported by the fact that trehalose protects nitrogenase from inactivation by oxygen (Jiménez-Zacarías et al., 2004). In another study (Suárez et al., 2008), a macroarray analysis of 7,200 expressed sequence tags from *P. vulgaris* nodules primed with *R. etli* overexpressing trehalose-6-phosphate synthase gene showed the upregulation of genes engaged in stress tolerance and carbon and N metabolism, suggesting a signaling mechanism for trehalose. Asafa et al. (2017) investigated the expression of three genes associated with stress responses and stress tolerance mechanisms in soybean plants by RT-PCR analyses. The study indicated an increased transcription of *GmDREBa* and *GmDREB2* (dehydration-responsive element-binding protein-type transcription factors) and *GmMYBJ1* (myeloblastosis transcription factor) in response to drought stress. However, inoculation of soybean plants with the trehalose-producing endophyte *Sphingomonas* sp. LK11 and exogenous trehalose application increased the mRNA gene expression of *GmDREBa*, *GmDREB2*, and *GmMYBJ1* as compared to the control plants, indicating trehalose-associated drought resistance. Taken together, these studies demonstrate that inoculation with endophytes and exogenous trehalose application enhanced soybean plant growth and stress tolerance. However, further work at the molecular level is needed to understand the role of bacterial trehalose in regulating plant genes. Moreover, no substantial work has been done on AMF-mediated abiotic stress tolerance through high trehalose accumulation. Under salinity stress, pigeon pea plants showed high trehalose accumulation in AMF-inoculated plants, which showed a high correlation between increased trehalose turnovers with increased nodulation. The higher accumulation of trehalose in AMF-mediated plants improved stability, in addition to enhancing nodulation and N fixation under salinity stress (Garg and Pandey, 2016).

From the abovementioned studies, it can be concluded that the manipulation of trehalose in microbes may be more promising for enhancing abiotic stress tolerance than altering the gene in plants *per se*. Microbes, particularly rhizobia, can produce larger amounts of trehalose (Streeter, 1985; McIntyre et al., 2007; Sugawara et al., 2010) and are stress responsive; thus, a constant production of trehalose by the plant would be unnecessary. Hence, focusing on the microbes would thus save plant resources, allowing crops to concentrate their energy on growth and reproduction.

## Conclusion and Future Directions

**FIGURE 2:**
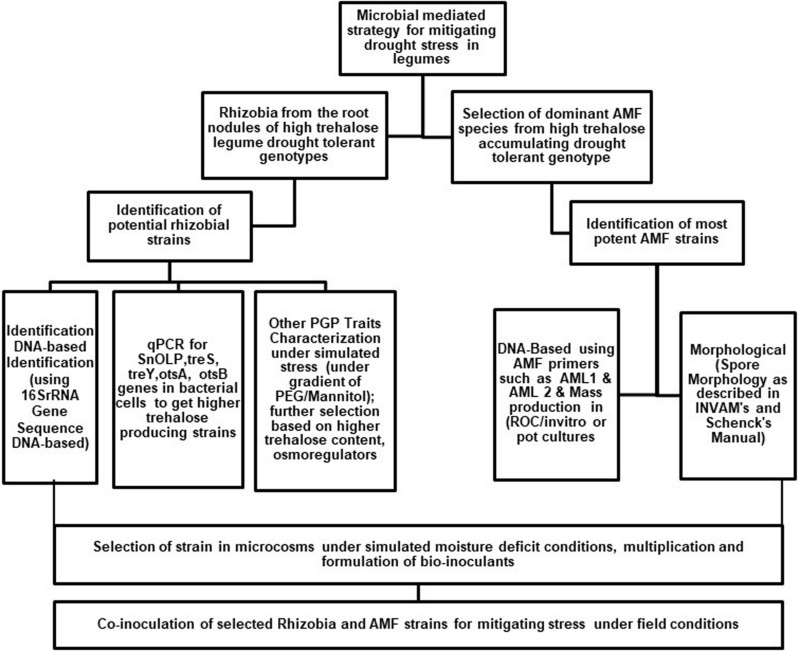
Diagrammatical representation for developing agro-microbial strategy utilizing arbuscular mycorrhizal fungi and rhizobia for mitigating drought stress in plants.

The importance of trehalose in improving stress tolerance, storage properties, and shelf life (Pereira et al., 2004) of microorganisms has been recognized in recent years and is being commercially exploited worldwide (McIntyre et al., 2007). Trehalose-over-producing microorganisms can enhance the tolerance of legume crops to abiotic stresses. However, the specific signaling mechanism behind the microbial trehalose-induced response to abiotic stress in plants requires further efforts. Indeed it is worthwhile to perform a detailed screening of plant varieties and breeding lines for having higher trehalose content in their root nodules during stress. At the same time, it is also promising to use the naturally colonizing native strains of rhizobia and AMF as starter cultures for breeding new lines capable of producing higher trehalose and improving stress tolerance. Increasing evidence indicates that plant trehalose application in plants, either through soil application or microbial inoculants, results in enhanced drought tolerance (Figure 2). However, the question of whether compatible solutes are equally efficient in protecting plants or bacteria from drought under field conditions needs further exploration. To conclude, we present a few critical research areas for enhancing stress tolerance in plants by microbe-mediated trehalose accumulation:

1. The selection of bacteria and bacterial endophytes capable of overproducing trehalose is very crucial. Exploring such bacteria for developing liquid inoculants with enhanced survival and stability is needed.
2. It is also vital to understand the role of trehalose metabolism in rhizobia through further screening of the trehalose synthase genes (e.g., *OtsA* gene) in the symbiotic bacteroids at the molecular, biochemical, and physiological levels to increase tolerance and grain yield under stress conditions (Suárez et al., 2008).
3. There is a strong need to carry out detailed investigations on the molecular mechanisms and omics-based approaches involved in AMF colonization during trehalose metabolism to confer tolerance in plants against abiotic stresses.

## Author Contributions

MPS created an outline, skeleton, and finalized the review for submission. MG compiled the work and contributed to the first draft. DC prepared figures and finalized the first draft based on the corrections made by other co-authors. AB contributed to collecting reviews and prepared references. RA formatted the manuscript text and figures in line to author guidelines. HM contributed to figures. AP, JB, SKS, LS, NM, SS-P, JG, and DJB provided intellectual inputs in the earlier drafts and in the finalization of the review. SKS corrected the drafts. All authors contributed to the article and approved the submitted version.

## Conflict of Interest

The authors declare that the research was conducted in the absence of any commercial or financial relationships that could be construed as a potential conflict of interest.
